# Autobrewery Syndrome and Endogenous Ethanol Production in Patients with MASLD: A Perspective from Chronic Liver Disease

**DOI:** 10.3390/ijms26157345

**Published:** 2025-07-30

**Authors:** Silvia Andaloro, Valeria De Gaetano, Ferdinando Cardone, Gianluca Ianiro, Lucia Cerrito, Maria Pallozzi, Leonardo Stella, Antonio Gasbarrini, Francesca Romana Ponziani

**Affiliations:** 1 Department of Translational Medicine and Surgery, Catholic University, 00168 Rome, Italy; silvia.andaloro01@icatt.it (S.A.); valeria.degaetano01@gmail.com (V.D.G.); luca.cardone27@gmail.com (F.C.); antonio.gasbarrini@unicatt.it (A.G.); 2 Liver Unit, CEMAD Centro Malattie dell’Apparato Digerente, Medicina Interna e Gastroenterologia, Fondazione Policlinico Universitario Agostino Gemelli IRCCS, 00168 Rome, Italy; gianluca.ianiro@policlinicogemelli.it (G.I.); lucia.cerrito@guest.policlinicogemelli.it (L.C.); mariapallozziucsc@gmail.com (M.P.); leonardo.stella@guest.policlinicogemelli.it (L.S.)

**Keywords:** autobrewery syndrome, gut microbiota, endogenous ethanol, chronic liver disease

## Abstract

Autobrewery syndrome is a rare condition characterized by the endogenous fermentation of carbohydrates by gut microbiota, which exceeds the liver’s detoxification capacity and leads to signs and symptoms of acute alcohol intoxication. This condition has significant clinical, social, and legal implications. Beyond the acute effects, the role of excessive endogenous ethanol production in the progression of chronic diseases—particularly liver disease—is still under investigation. In this review, we aim to describe the key clinical features of autobrewery syndrome, identify the main microbial pathogens involved, and explore the potential impact of endogenous ethanol production on the development and progression of chronic liver disease. Although robust data and standardized treatment protocols are currently lacking, we discuss the general principles of management and outline possible therapeutic strategies and future perspectives.

## 1. Introduction

Auto-brewery syndrome (ABS), also known as gut fermentation syndrome, is a rare and likely underdiagnosed condition in which specific gut microorganisms produce excessive amounts of ethanol via the fermentation of carbohydrates. This endogenous ethanol production may result in clinical signs and symptoms of alcohol intoxication. Despite its rarity, ABS can have substantial clinical, social, and legal implications, and physicians should be aware of its existence. For instance, patients with ABS may be erroneously excluded from organ transplant programs or may test falsely positive for alcohol during routine checks in addiction treatment programs [1]. There are also case reports of ABS being diagnosed following positive traffic alcohol tests, including in the context of motor vehicle accidents [2,3]. However, the applicability of ABS as a legal defense remains controversial [4].

Fewer than 100 cases of ABS have been reported in the medical literature since it was first described in 1952, and the majority of published reports are isolated case studies, with a paucity of systematic investigations. Moreover, the validity of some reported cases is limited by the inability to completely exclude exogenous alcohol intake and by inconsistencies in diagnostic procedures, emphasizing the need for standardized testing protocols. Table 1 provides a summary of the main clinical reports to date. The earliest case, described in 1948, involved a five-year-old child in Africa [5], and subsequent reports have documented ABS in both adults and children, of various sexes and nationalities [6,7,8].

Although ABS can occur in otherwise healthy individuals, certain predisposing factors have been identified. These include prior antibiotic use, which may disrupt gut microbial balance and favor the overgrowth of fermentative organisms [9], as well as underlying conditions such as diabetes mellitus [10], Crohn’s disease [11], short bowel syndrome [12], chronic intestinal pseudo-obstruction [13], and Roux-en-Y gastric bypass surgery [14]. A common feature among these conditions is impaired small intestinal function, which can result in carbohydrate malabsorption and promote ethanol production by fermenting gut microbiota [6].

As previously noted, endogenous fermentation of carbohydrates by gut microorganisms is central to the pathogenesis of ABS. Although the condition is typically associated with the gastrointestinal tract, rare cases of urinary and oral fermentation have also been reported [15].

The primary microbial agents implicated in ABS are yeasts, particularly *Saccharomyces cerevisiae* and *Candida* spp. [3,16]. However, bacterial species have also been implicated. For example, *Klebsiella pneumoniae* strains with high ethanol-producing capacity have been identified in patients with metabolic dysfunction-associated steatotic liver disease (MASLD) [17], and *Escherichia coli* has also been reported as a possible contributor [18]. More recently, additional bacterial and fungal species have been proposed as causative agents, reflecting the growing recognition of the complex role played by the gut microbiome in ABS.

While the acute manifestations and social-legal implications of ABS are increasingly recognized, the impact of endogenous ethanol production on chronic liver disease and cirrhosis remains largely unexplored. Additionally, the contribution of gut microbiota dysbiosis to ethanol production warrants further investigation.

This review aims to summarize the alterations in gut microbial composition observed in ABS, assess the role of endogenous ethanol in liver disease pathogenesis and progression, and discuss current and potential therapeutic strategies.

**Table 1:** Summary of the main clinical cases of auto-brewery syndrome (ABS) reported in the literature. The table includes key patient characteristics, along with clinical manifestations, the microorganisms isolated from fecal samples or duodenal aspirates, treatment and outcome. Abbreviations: NA, not available.

Author	Age	Sex	Comorbidities	Symptoms	Microorganism	Blood Ethanol Level	Treatment	Outcome
Kaji et al. [19]	1. 24 2. 35	1. F 2. M	1. None 2. None	1. Nausea, faintness 2. Slurred speech, blurred vision, balance problems	1. *C. albicans* and *C. krusei* 2. *C. albicans*	1. 254 mg/dL 2. NA	1.Cabimicina, laxatives, carbohydrates restriction 2.Nystatinum	1. Symptoms resolution 2. Symptoms resolution
Spinucci et al. [13]	44	M	Chronic intestinal pseudo-obstruction, on long-term home parenteral nutrition	Abdominal pain, bloating, disorientation, slurred speech after recent antibiotic therapy and simple-sugar based diet	*C. albicans* (small bowel cultures and stool) *S. cerevisiae* (small bowel cultures and stool)	24.9 mmol/L	Sugar-free diet + fluconazole	Symptoms resolution, normalization of ethanolemia
Welch et al. [11]	71	M	Crohn’s disease, small bowel resection	Slurred speech, dizziness after receiving antibiotic therapy and increasing sugar intake	*C. glabrata* (small bowel cultures)	234 mg/dL	Low carbohydrate diet, avoiding antibiotics	Symptoms resolution, normalization of ethanol levels
Vandekerckhove et al. [20]	47	M	Roux-en-J bypass	Dizziness after receiving antibiotic therapy	*Candida glabrata* (stool)	34.7 mmol/L	Low carbohydrate diet + fluconazole nystatin; amphotericin; FMT	Diet and antifungal unsuccessful; FMT symptoms resolution, normalization of ethanol levels and liver enzymes
Dahshan and Donovan [21]	13	F	Short bowel syndrome in jejunal atresia, 5–6 UA per week	Disorientation, somnolence, bizarre behaviour, fruity odor of breath, especially after meals	*C. glabrata* (small bowel cultures) *S. cerevisiae* (small bowel cultures)	250–350 mg/dL	Antifungal (fluconazole)	Symptoms resolution, normalization of ethanol levels
Saverimuttu et al. [22]	45	M	Diabetes mellitus type 2, hypertension, hyperlipidemia	Seizures, slurred speech, poor coordination related to meal intake, after receiving antibiotic therapy	*S. cerevisiae* (stool) *C. intermedia* (small bowel cultures) *K. pneumoniae* (small bowel cultures) *E. faecalis* (small bowel cultures)	410 mg/dL	Low carbohydrate diet, antifungal (micafungin), probiotics	Symptoms resolution, normalization of ethanol levels
Malik et al. [23]	46	M	None	Memory loss, mood changes, depression after receiving antibiotic therapy	*S. Cerevisiae* (stool) *S. boulardii* (stool) *C. Albicans* (small bowel cultures) *C. parapsilosis* (small bowel cultures)	200 mg/dL at admission, up to 400 mg/dL	Carbohydrate-free diet, multiple antifungal therapy, probiotics	Symptoms resolution
Ser et al. [24]	58	F	Hemicolectomy, herpetic encephalitis	Recurrent encephalopathy episodes, chronic cognitive disturbances, carbohydrates craving, fruity odor of breath	*C. krusei* (stool) *C. parapsilosis* (stool)	315 mg/dL	Low carbohydrate diet, antifungal (nystatin)	Symptoms resolution
Jansson-Nettelbladt et al. [25]	3	F	Small bowel malformation	Balance problems	*C. kefyr* (small bowel cultures and stool) *S. cerevisiae* (small bowel cultures)	15 mmol/L	Low carbohydrate diet, antifungal (fluconazole)	Symptoms resolution
Kruckenberg et al. [1]	61	F	Poorly controlled diabetes mellitus, cirrhosis	None	*C. glabrata* (urinary) *S. cerevisiae* (urinary)	Urine ethanol level 32 mg/dL	Antifungal regimen	None improvement of urine ethanol levels
Gruszecki et al. [26]	19	F	Diabetes mellitus type 1	Severe diabetic ketoacidosis	*C. glabrata* (urinary)	Urine ethanol level 0.32 g/dL	NA	Death
Cordell et al. [27]	1. 60 2. 42 3. 32	1. M 2. F 3. M	1. Alcohol abuse; hepatitis C, hypertension, pre-diabetes 2. None 3. None	1. drunkenness 2. drunkenness, loss of coordination 3. Abdominal pain, reflux, diarrhea, nausea	1. *C. albicans, C. krusei* (stool) 2. *S. cerevesiae* (stool), *S. bulardii* (stool) 3. None	1. 170 mg/dL 2. 0.40% on breathalyzer = 400 mg/dL 3. NA	Low-carbohydrate diet Diet NA	Symptoms relief Symptoms relief NA
Yuan et al. [17]	NA	NA	NA	NA	*K. pneumoniae* high-alcohol-producing (HiAlc Kpn) (stool)	∼400 mg/dL	NA	Oral gavage of HiAlc Kpn and FMT with HiAlc *Kpn* induced MASLD
Akbaba et al. [2]	38	M	Recently stopped alcohol abuse, sleep disorders, hypertension	Screening after a car accident	*Pseudomonas* (small bowel cultures)	322 mg/dL	NA	NA
Yates and Saito [28]	52	M	Recent SARS-CoV2 infection	Dizziness, slurred speech, behavior changes	None	212 mg/dL	Low-carbohydrate diet, probiotics, fluconazole	Symptoms relief
Akhavan et al. [29]	25	M	Recently undertaken ketogenic diet	Slurred speech, stumbling, dizziness, nausea	None, but resolved after empiric fluconazole therapy	0.3 g/dL	Empiric fluconazole	Symptoms resolution

### 1.1. General Approach to the Autobrewery Syndrome

#### 1.1.1. Endogenous Ethanol Production

Under physiological conditions, a small amount of ethanol is constantly produced endogenously, primarily as a byproduct of microbial metabolism in the gut [30,31,32,33]. Several studies have demonstrated that intestinal microorganisms, particularly yeasts and fermentative bacteria, are capable of producing ethanol when cultivated in the presence of various carbohydrates [34].

Ethanol may also arise non-microbiologically through the oxidation of acetaldehyde, which itself is generated during normal metabolism of substrates such as pyruvate, threonine, deoxyribose-5-phosphate, phosphoethanolamine, and alanine. Acetaldehyde can be further oxidized to acetate by aldehyde dehydrogenase (ALDH), present in both colonic mucosal cells and intestinal bacteria. Alternatively, it may be absorbed into portal circulation, especially under conditions of impaired ALDH activity [35,36]. When hepatic ALDH function is compromised, intracellular levels of acetaldehyde may rise, favoring its reduction back to ethanol via a reversible NAD^+^/NADH-dependent reaction catalyzed by alcohol dehydrogenase (ADH) [37].

In healthy individuals, endogenous ethanol production is clinically irrelevant, with blood concentrations typically measured in the range of 0.01–0.02 mg/dL [4]. Moreover, hepatic ADH activity ensures rapid and efficient clearance of ethanol, following Michaelis-Menten elimination kinetics [38,39]. However, this balance may be disrupted under certain pathological conditions. Gut dysbiosis, characterized by fungal overgrowth or an increase in ethanol-producing bacteria, combined with impaired hepatic ethanol metabolism, can lead to accumulation of ethanol in the systemic circulation, as reported in cases of short bowel syndrome [37]. Although yeast species have shown a significant capacity for ethanol production in vitro, the extent to which similar levels occur in vivo remains uncertain. Reaching blood ethanol concentrations comparable to those observed in ABS would likely require a substantial microbial load operating under optimal fermentation conditions—factors that have not yet been rigorously evaluated in clinical settings.

Although data on endogenous ethanol concentrations in patients with liver cirrhosis are limited and inconclusive, some studies suggest that altered gut microbiota, bacterial overgrowth, and impaired intestinal motility may contribute to higher systemic ethanol levels in this population [40]. In addition, it has been hypothesized that increased microbial ethanol levels in some patients may result from insulin-mediated suppression of ADH activity rather than from excessive production per se, although this remains to be confirmed. Ethanol produced by intestinal microorganisms is absorbed into the portal circulation and undergoes the hepatic first pass; when saturating the liver detoxification capacity, symptoms of ABS can occur.

#### 1.1.2. ABS Clinical Presentation

**Figure 1:**
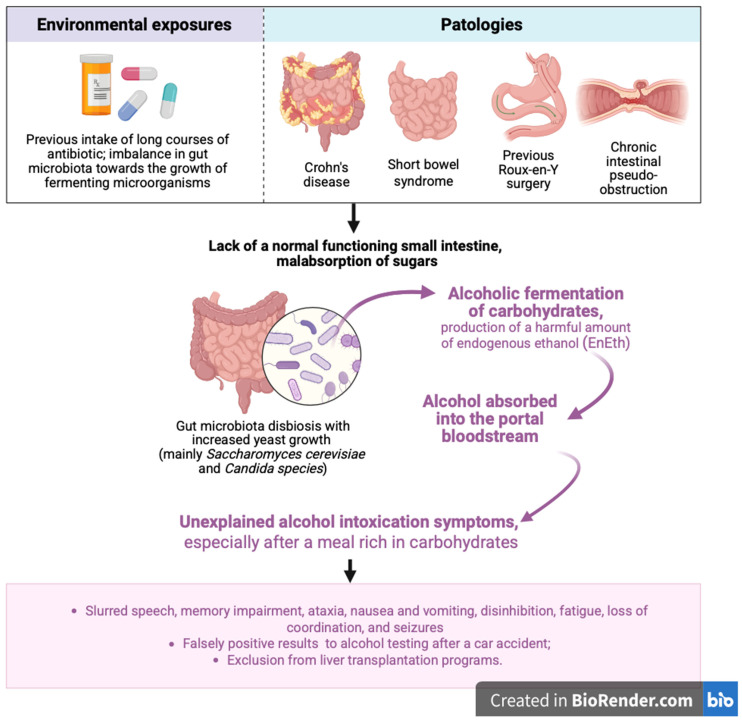
Overview of the main risk factors involved in the development of alcoholic carbohydrate fermentation, along with key steps in the pathogenesis and clinical manifestations of auto-brewery syndrome (ABS). EnEth = endogenous ethanol. Figure created with BioRender.com (accessed on 26 July 2025).

The clinical presentation resembles that of exogenous alcohol intoxication—especially after carbohydrate-rich meals—and may include slurred speech, memory impairment, ataxia, nausea and vomiting, disinhibition, fatigue, loss of coordination, and seizures (Figure 1).

Due to the rarity of the condition and its complex clinical, social, and legal implications, diagnosis requires careful exclusion of alternative causes and, most importantly, covert alcohol consumption. A thorough patient history—focusing on potential risk factors and prior unexplained episodes of alcohol-like symptoms—should be followed by a physical examination. Laboratory testing is essential. Blood, breath, and urine alcohol levels should be measured and correlated with symptoms. Stool cultures may also be performed, though the presence of fermentative yeasts in the lower gastrointestinal tract may not be clinically significant [41]. Therefore, upper endoscopy is preferred for microbiological sampling, as it allows collection from the stomach and small intestine and facilitates antimicrobial susceptibility testing.

The gold standard for diagnosis is the glucose challenge test, which involves monitoring blood and breath alcohol levels at baseline and at 2, 4, 8, 16, and 24 h after ingestion of 100–200 g of glucose. This test must be performed under medical supervision to ensure no exogenous alcohol intake. In affected individuals, glucose is rapidly fermented into ethanol, whereas no significant alcohol level increase is observed in healthy controls.

Management of acute alcohol intoxication follows standard supportive measures: intravenous hydration, airway protection, correction of nutritional deficiencies (e.g., folate and thiamine) [41], and treatment of complications. Withdrawal symptoms, when present, should be managed similarly to alcohol dependence.

Treatment of ABS focuses on a low-carbohydrate diet, antifungal or antibacterial therapy based on the identified pathogen, and probiotics as adjunctive therapy. Malik, Wickremesinghe, and Saleem proposed a structured treatment protocol that includes a 6-week course of a low-carbohydrate diet plus antifungals (first-line: nystatin; second-line: oral azoles; third-line: IV micafungin), twice-daily monitoring of breath alcohol concentration (BAC), and gradual tapering of antifungal therapy over an additional 6 weeks following symptom resolution and negative BAC.

The use of probiotics, particularly *Lactobacillus* spp., may help suppress *Candida albicans* by inhibiting biofilm formation and yeast filamentation [23,42,43]. However, their clinical efficacy remains uncertain.

An emerging area of interest is fecal microbiota transplantation (FMT) [44]. A case report [20] described successful treatment of ABS with FMT following failure of conventional therapies.

## 2. The Intricacy of the Composition of the Microbiota and Its Metabolic Role

Millions of microorganisms from diverse species inhabit the human gastrointestinal tract. These are predominantly bacteria, especially anaerobic species [45], while fungi (*Candida, Saccharomyces*, and *Cladosporium*) and viruses are present to a lesser extent, forming approximately 2% of all gut microbes [46]. Other microbial residents include phages, protozoa, and archaea [47].

The gut microbiome is a dynamic ecosystem whose composition is modulated by multiple factors, including genetics, nutrition, mode of delivery, breastfeeding, infections, antibiotic exposure, and circulating pathogens [48]. Microbiota composition varies not only across populations [49], but also among individuals within the same population and even throughout the lifespan of a single individual [50].

Alterations in the gut microbiota, known as dysbiosis, are implicated in the pathogenesis of various diseases. Metagenomic analyses have revealed significant shifts in microbial composition in metabolic syndrome (p. 5, [51]), metabolic dysfunction-associated steatohepatitis (MASH), and liver cirrhosis [52,53], often associated with increased production of metabolic and inflammatory mediators [54]. Changes in mycobiome composition have also been linked to gastrointestinal diseases such as ulcerative colitis [55] and Crohn’s disease [56].

Beyond the gastrointestinal tract, microbiota alterations have been associated with extraintestinal conditions, including psychiatric disorders such as major depressive disorder and bipolar disorder [57], and allergic asthma [58], suggesting that gut dysbiosis may modulate immune responses in distant organs.

Increased intestinal permeability has been associated with microbiota alterations [59]. In parallel, changes in the mycobiome may contribute to epithelial barrier damage, promoting the translocation of fungal components and immune cells [60]. The composition of the gut microbiota also plays a fundamental role in metabolic homeostasis and immune regulation through the production of peptides and microbial-derived bioactive compounds with immunomodulatory properties [61].

### Alterations in the Composition of the Gut Microbiota in Autobrewery Syndrome

During digestion, small amounts of endogenous ethanol (EnEth) can be produced through fermentation of carbohydrates by certain bacteria and fungi that are part of the normal gut microbiota [62]. Under physiological conditions, this process is self-limited. However, in the setting of intestinal dysbiosis, often following prolonged antibiotic therapy, some commensal ethanol-producing microorganisms may become opportunistic pathogens, leading to abnormally elevated ethanol production and detectable blood ethanol levels.

Among the microbial agents implicated in ABS, yeasts are the most frequently involved [3]. Most yeasts can ferment sugars anaerobically. Interestingly, species such as *Candida glabrata* and *Saccharomyces cerevisiae* can also ferment sugars in the presence of oxygen—an evolutionary adaptation known as the Crabtree effect—which enables them to suppress the growth of competing organisms [63,64]. A study by Bivin and Heinen [16] demonstrated that *C. albicans, C. tropicalis, S. cerevisiae*, and *Torulopsis glabrata* were capable of ethanol production when incubated with various carbohydrate-rich infant formulas over 24–48 h.

To identify the microorganisms involved in ABS, several studies have analyzed small intestinal colonization, the primary site of ethanol absorption. Microbes have been isolated through stool cultures, glucose challenge testing, and endoscopic aspiration of gastric, small bowel, and colonic fluids. The most commonly isolated fungal species were *C. albicans* [13,19], *C. parapsilosis* [23,24], *C. glabrata* [11,20,21], *C. intermedia* [22], *C. kefyr* [25], *C. krusei* [24], *Saccharomyces cerevisiae* [13,21,22], and *S. boulardii* [23]. Both *S. cerevisiae* and *C. albicans* are organisms that optimally proliferate at a pH of 4–6 [65,66]. The use of antacids may promote fungal growth and facilitate ethanol synthesis in the presence of carbohydrates [67].

Among bacteria, *Klebsiella pneumoniae* appears to be a significant ethanol producer [68]. A study by Yuan et al. [17] isolated a high-alcohol-producing strain (HiAlc-Kpn), which was associated with metabolic-dysfunction-associated steatotic liver disease (MASLD) in 60% of the study population. Oral administration of this strain to mice induced hepatic steatosis. Similar findings were confirmed in studies by Li et al. [69] and Xue et al. [70], demonstrating ethanol production in vivo in both mice and rabbits. In a case reported by Akbaba et al. [2], *Pseudomonas* spp. were isolated from duodenal aspirates, although this genus is not a typical component of the human gut microbiota.

Interestingly, SARS-CoV-2 infection may also play a role in altering gut microbial composition. A case of ABS was reported shortly after COVID-19 [28], and broader studies have shown that SARS-CoV-2-positive patients exhibit a reduction in beneficial commensals and increased levels of opportunistic pathogens [71].

Beyond the gastrointestinal tract, extraintestinal manifestations of ABS have been described: urinary auto-brewery syndrome has been reported in patients with poorly controlled diabetes, where sugar fermentation by *C. glabrata* occurs within the bladder, again via the Crabtree effect [1,26].

Oral ethanol fermentation has been demonstrated in the oral cavity and periodontal tissues [15,72]. The intermediate metabolite acetaldehyde, poorly cleared due to low oral acetaldehyde dehydrogenase activity, can persist and increase the risk of oral squamous cell carcinoma [73].

## 3. Alcohol-Mediated Liver Damage and Endogenous Ethanol in Chronic Liver Disease

### 3.1. Metabolism of Alcohol and Alcohol Mediated Liver Damage

In the liver, ethanol is metabolized through oxidative and non-oxidative pathways. The oxidative metabolism, which accounts for the majority of ethanol degradation, involves two key enzyme systems, alcohol dehydrogenase (ADH) and the microsomal ethanol oxidizing system (MEOS).

Ethanol is primarily metabolized in the liver through oxidative pathways involving ADH [39] and cytochrome P450 2E1 (CYP2E1), with catalase playing a minimal role. These enzymes convert ethanol into acetaldehyde, a toxic intermediate that contributes to oxidative stress, liver injury, and inflammation [74].

The non-oxidative pathway, although quantitatively limited, leads to the formation of compounds such as fatty acid ethyl esters (FAEEs), phosphatidylethanol (PEth), ethyl glucuronide (EtG), and ethyl sulfate (EtS) [75,76]. These metabolites are relevant biomarkers for alcohol intake.

#### 3.1.1. Lipid Accumulation, Mitochondrial Dysfunction and Oxidative Stress

Ethanol metabolism profoundly alters the hepatic redox state, notably by decreasing the NAD^+^/NADH ratio [77,78], which impacts several metabolic pathways, including inhibition of glycolysis, the citric acid cycle, pyruvate dehydrogenase, fatty acid β-oxidation, and gluconeogenesis [39]. Moreover, the accumulation of acetaldehyde—a highly reactive and toxic metabolite—contributes to hepatic steatosis, inflammation, fibrosis, and carcinogenesis.

Acetaldehyde disrupts lipid metabolism by impairing mitochondrial β-oxidation and enhancing lipogenesis, leading to elevated circulating free fatty acids (FFAs) [79] and hepatic steatosis through multiple transcriptional and enzymatic pathways [80,81]. Moreover, it inhibits AMPK signaling and upregulates lipogenic enzymes and transcription factors, thereby promoting hepatic lipid synthesis and triglyceride accumulation [82,83,84].

One of the key contributors to hepatic steatosis is impaired mitochondrial fatty acid β-oxidation, considered the most significant metabolic disturbance in alcohol-related liver disease [85]. Ethanol accumulation inhibits sirtuin 1 (SIRT1) activity [86], which increases the LIPIN1β/α ratio, shifting LIPIN1 function toward lipid synthesis and away from mitochondrial fatty acid oxidation. Additionally, acetaldehyde impairs peroxisome proliferator-activated receptor alpha (PPARα)—a key regulator of mitochondrial β-oxidation—by promoting its inactivation [87,88]. Finally, acetaldehyde can form covalent adducts with proteins, DNA, and lipids by reacting with amino, hydroxyl, and sulfhydryl groups. These adducts cause direct hepatocyte injury, mitochondrial dysfunction, and genomic instability [89]. As acetaldehyde metabolism increases mitochondrial oxygen consumption to regenerate NAD^+^, ROS are generated as a byproduct [90]. Excessive ROS leads to lipid peroxidation, enzyme inactivation, DNA damage, and membrane disruption, thereby amplifying inflammatory responses and promoting hepatocarcinogenesis [91].

#### 3.1.2. Intestinal Barrier Impairment

It has been extensively demonstrated that chronic alcohol consumption disrupts the intestinal epithelial barrier, particularly affecting the integrity of adherens junctions (AJs) and tight junctions (TJs) in the colonic mucosa [92,93]. One of the key mechanisms involves acetaldehyde, which impairs barrier function by inhibiting protein tyrosine phosphatase (PTPase) activity. This inhibition leads to hyperphosphorylation of ZO-1, E-cadherin, and β-catenin [94], disrupting the intracellular interaction between β-catenin and E-cadherin. Consequently, this also compromises the extracellular adherence functions of E-cadherin, ultimately resulting in adherens junction disassembly. Loss of AJs leads secondarily to disruption of tight junctions [95]. Furthermore, ethanol has been shown to alter cytoskeletal structures, including actin filaments and microtubules, through the activation of myosin light chain kinase (MLCK) [96], and to induce nitric oxide (NO) production [97], both of which further compromise tight junction integrity. As a consequence, the translocation of microbial products—particularly lipopolysaccharide (LPS)—from the gut lumen into the circulation is promoted. This triggers Kupffer cell activation, resulting in ROS production and the release of chemokines that recruit bone-marrow-derived neutrophils and circulating monocytes into the liver [79,98,99]. Subsequently, activation of the Toll-like receptor 4 (TLR4)/Mitogen-activated protein kinase (MAPK)/nuclear factor-kappa B (NF-κB) signaling axis occurs, promoting the expression of pro-inflammatory cytokines such as tumor necrosis factor alpha (TNF-α) and interleukin-1β (IL-1β) [100,101,102].

#### 3.1.3. Hepatic Stellate Cell Activation and Liver Fibrogenesis

Activation of hepatic stellate cells (HSCs) is a pivotal event in the progression of chronic liver injury to fibrosis and cirrhosis. Upon liver injury, quiescent HSCs differentiate into myofibroblast-like cells, characterized by increased proliferation, cytokine production, and extracellular matrix (ECM) deposition, notably type I collagen [91].

**Figure 2:**
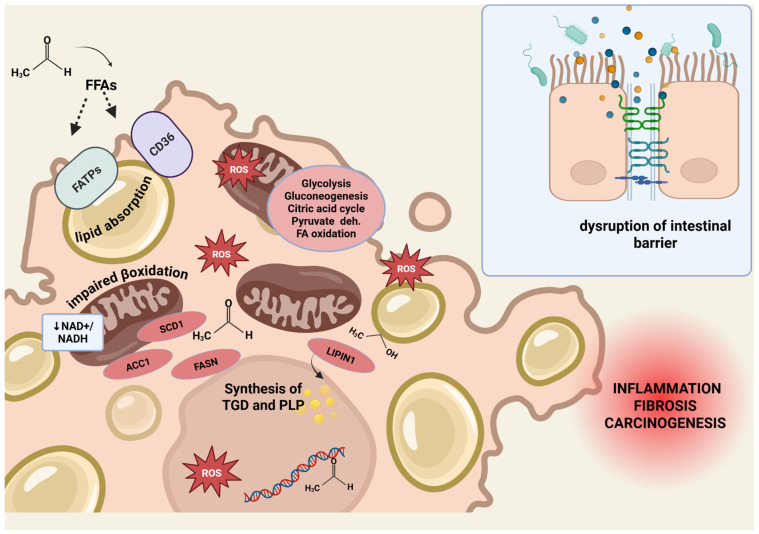
Harmful effect of alcohol metabolism. Harmful effects of alcohol metabolism on hepatic lipid homeostasis and intestinal barrier integrity. Ethanol and its metabolite acetaldehyde contribute to lipid accumulation and liver injury through multiple mechanisms. Enhanced lipid uptake: Acetaldehyde increases the release of free fatty acids (FFAs) from adipose tissue and promotes hepatic uptake via upregulation of fatty acid transport proteins (FATPs) and FAT/CD36. Stimulation of lipid synthesis: Acetaldehyde upregulates lipogenic genes, including acetyl-CoA carboxylase 1 (ACC1), fatty acid synthase (FASN), and sterol-CoA desaturase 1 (SCD1). Ethanol induces LIPIN1, promoting triglyceride and phospholipid synthesis. Impaired lipid clearance: Altered cellular redox balance due to ethanol and acetaldehyde metabolism leads to mitochondrial dysfunction, impairing β-oxidation. Production of reactive oxygen species (ROS) causes lipid peroxidation, enzyme inactivation, DNA damage, and cell membrane disruption, fostering inflammation and carcinogenesis. Chronic alcohol consumption disrupts the intestinal epithelial barrier by impairing adherens and tight junctions, promoting LPS translocation and liver inflammation via TLR4/NF-κB signaling and pro-inflammatory cytokine release. Abbreviations: ACC1, acetyl-CoA carboxylase 1; FFAs, free fatty acids; FASN, fatty acid synthase; FATPs, fatty acid transport proteins; ROS, reactive oxygen species; SCD1, sterol-CoA desaturase 1. Figure created with BioRender.com.

Acetaldehyde plays a central role in alcohol-induced liver fibrosis (Figure 2), promoting de novo collagen synthesis and ECM remodeling [103,104]. In human HSCs, acetaldehyde activates intracellular signaling pathways that promote the transcription of fibrogenic genes, such as collagen type I alpha 2 chain (COL1A2) and fibronectin. [105]. It also activates the TGF-β/SMAD3 signaling pathway, which enhances the production of fibrillar collagens and structural glycoproteins, further contributing to fibrogenesis [106]. Additionally, acetaldehyde contributes to ECM remodeling by upregulating matrix metalloproteinase-2 (MMP-2) and downregulating MMP-1, thereby shifting ECM turnover toward fibrotic accumulation [107,108]. Experimental models further support a strong association between CYP2E1 activity, oxidative stress, and the progression of fibrosis in alcohol-induced liver injury [109,110].

Although this section outlines general mechanisms of alcoholic liver disease (ALD), it is important to note that most published cases of ABS do not include detailed information on whether ALD-related pathological changes—such as steatosis, alcoholic hepatitis, or fibrosis—were present. Regarding previous cases of ABS mentioned above, an increase in liver enzymes and the presence of hepatomegaly or hepatic steatosis were reported in some instances [2,13,20], with normalization of liver enzymes following the normalization of blood ethanol levels [20]. However, since the effect of ABS on liver function was not the primary focus of these reports, a thorough diagnostic workup and/or longitudinal follow-up regarding liver disease was generally lacking. Moreover, due to the relatively short observation periods—primarily centered around symptoms caused by acute alcohol intoxication—the potential long-term impact of chronic endogenous ethanol production in these patients has not been investigated. A few reported cases involved comorbidities such as Hepatitis C and a history of alcohol use disorder [27], or diabetes and liver cirrhosis [1,26], where a more in-depth hepatic evaluation would have been particularly informative. Overall, systematic assessment of ALD features in ABS patients remains lacking, representing a significant gap in the literature and a potential direction for future research.

## 4. Autobrewery Syndrome, MASLD, and Chronic Liver Disease

As previously discussed, ABS is a rare clinical condition that may be associated with other comorbidities. Although ABS and physiological endogenous ethanol production are distinct clinical entities, they may share overlapping mechanistic features, particularly in the context of MASLD, where low-level but persistent microbial ethanol production may contribute to liver injury, even in the absence of overt signs of intoxication. Despite involving the overproduction of endogenous ethanol, which may in theory contribute to liver damage, robust clinical evidence linking ABS to chronic liver disease is still limited.

In a study by Hafez et al. [10], patients with liver cirrhosis or diabetes mellitus demonstrated significantly higher blood alcohol concentrations following ingestion of 75 g of glucose compared to healthy controls. The effect was even more pronounced in patients presenting with both conditions. These findings suggest that endogenous ethanol production can occur not only in ABS, but also in healthy individuals, likely mediated by the gut microbiota, as supported by postprandial increases in blood ethanol levels despite the absence of dietary ethanol intake.

Under normal conditions, hepatic ADH effectively metabolizes small amounts of endogenous ethanol. This is evidenced by the elevation in blood ethanol levels following pharmacological inhibition of ADH using 4-methylpyrazole [30,111]. In recent years, growing attention has been directed toward the interplay between ABS, endogenous ethanol production, and chronic liver diseases, especially those within the metabolic-associated spectrum, such as MASLD and MASH. For instance, Volynets et al. [112] demonstrated that patients with MASLD exhibit increased endogenous ethanol synthesis and altered intestinal permeability, which may heighten hepatic exposure to microbial metabolites, including ethanol.

In MASLD, HiAlc-Kpn was shown to induce hepatic steatosis in germ-free mice, leading to elevated serum ALT and AST levels, hepatic triglyceride and thiobarbituric acid reactive substance TBARS accumulation, and upregulation of lipogenesis and PPARα gene expression [17]. Interestingly, histological similarities between MASLD and alcoholic hepatitis—including hepatic steatosis and Mallory-Denk bodies—have long raised the hypothesis of a shared pathogenesis, with endogenous alcohol representing the internal counterpart of exogenous alcohol in MASH [113]. This idea is supported by findings of increased expression of alcohol-metabolizing enzymes in liver tissue from MASH patients without alcohol intake [114]. Zhu et al. [111] further reported elevated blood ethanol concentrations in obese patients with MASH, but not in obese patients without MASH. Moreover, they described distinct gut microbiota profiles, with MASH patients harboring ethanol-producing E. coli strains. Similarly, Meijnikman et al. [115] detected higher ethanol concentrations in portal vein blood from MASLD patients compared to controls without steatosis—demonstrating a more direct link between microbial ethanol production and hepatic exposure. Beyond metabolic effects, endogenous ethanol has been shown to induce mitochondrial dysfunction in MASH, reducing ATP levels, increasing ROS accumulation, and promoting mitochondrial DNA damage—factors known to contribute to inflammation and fibrogenesis [116].

Taken together, these findings suggest a potential pathophysiological role of ethanol-producing gut microbiota in the development and progression of MASH and cirrhosis. While the liver is equipped with a powerful capacity for ethanol detoxification, in patients with advanced liver disease or ABS, the burden of microbial ethanol production may exceed the hepatic clearance threshold, contributing to ongoing liver injury.

## 5. Treatment and Future Perspectives of Autobrewery Syndrome

Due to its rarity and the limited understanding of its pathophysiology, robust data on effective treatment strategies for ABS remain scarce. To date, no case-control studies have been conducted, and available evidence is based exclusively on a small number of case reports. Nevertheless, given the underlying mechanisms of ABS, potential therapeutic strategies may involve modulation of hepatic alcohol metabolism and targeted regulation of the gut microbiota.

Recent experimental studies have highlighted several potential therapeutic targets for ALD, which may have translational relevance for ABS. For instance, the methylation-controlled J protein (MCJ)—an intrinsic inhibitor of mitochondrial respiration—has recently been identified as a key mediator in ALD progression. In early stages of liver injury, MCJ expression is reduced, but it increases as damage progresses. Hepatic silencing of MCJ improved mitochondrial function without inducing oxidative stress, suggesting a novel therapeutic approach to mitigate alcohol-induced hepatotoxicity [117]. Similarly, the activation of Toll-like receptor 7 (TLR7) with an oral agonist could protect against ethanol-induced liver injury by preserving the intestinal barrier, reducing bacterial translocation and attenuating hepatic inflammation and steatosis [118]. In a murine model of alcohol and LPS-induced liver injury, omega-3 polyunsaturated fatty acid (n-3 PUFA) supplementation was shown to attenuate hepatic damage, supporting its potential role as a nutritional intervention in alcohol-related liver disease [119].

Given the pivotal role of the gut microbiome in ABS pathogenesis, several strategies have been proposed to reduce ethanol-producing microorganisms and restore eubiosis. Antifungal therapy, particularly fluconazole, combined with a low-carbohydrate diet, has been effective in several ABS case reports [3]. In some instances, patients required escalation to alternative antifungal agents. Treatment selection should ideally be guided by microbial identification and susceptibility testing.

Antibiotics, including fluoroquinolones and carbapenems, have shown a significant reduction in bacterial ethanol production in both experimental models and clinical reports. These agents are often administered alongside low-carbohydrate diets and probiotics, such as *Lactobacillus*, *Clostridium butyricum*, and *Bacillus* spp. [32]. Rifaximin, a non-absorbable broad-spectrum antibiotic, exerts its activity within the gastrointestinal tract and is already used in the treatment of hepatic encephalopathy. Although its regulatory effect on the gut microbiome offers a plausible rationale for use in ABS, this indication remains speculative. Given the rising concern over antimicrobial resistance, the use of antibiotics should be reserved for carefully selected cases and preceded by microbiological confirmation.

Probiotics represent a safe and non-invasive strategy for microbiota modulation. *Lactobacillus rhamnosus* GG (LGG) has demonstrated protective effects against ethanol-induced liver injury in experimental settings, primarily by enhancing intestinal barrier integrity through the release of AhR ligand-enriched extracellular vesicles, which upregulate regenerating islet-derived protein 3 gamma (Reg3) and Nuclear Factor Erythroid 2-related factor 2 (Nrf2) expression [120].

In addition, phage therapy has been investigated as an alternative to antibiotic therapy to treat HiAlc-Kpn [68]. In male mice with HiAlc Kpn-induced steatohepatitis, treatment with HiAlc Kpn-specific phage was able to alleviate liver damage by regulating inflammation and basal metabolism, without significant relevant changes in the microbiota.

Although data remain extremely limited, fecal microbiota transplantation (FMT) has emerged as a promising option for microbiota restoration in ABS. To date, Vandekerckhove et al. [20] reported the only documented case of successful treatment of ABS with FMT following failure of standard antifungal therapy.

Beyond ABS, clinical trials have shown that FMT can induce favorable shifts in gut microbiota composition and function in patients with alcohol-associated hepatitis [121,122,123]. Preclinical models have also demonstrated their potential benefit in MASLD, notably improving intestinal permeability and reducing hepatic inflammation [124,125].

In summary, despite the lack of standardized therapeutic guidelines, emerging experimental and clinical data suggest that targeting gut microbiota, supporting liver mitochondrial function, and modulating innate immune responses may offer promising strategies for the management of ABS and related liver conditions. However, larger controlled studies are urgently required to validate these findings, clarify the most effective interventions, and develop evidence-based clinical algorithms for the diagnosis and treatment of this complex and underrecognized syndrome.

## 6. Conclusions

ABS is a rare and likely underdiagnosed condition, characterized by pathological endogenous ethanol production resulting from gut dysbiosis and the overgrowth of ethanol-producing microorganisms. Chronic liver disease, which is strongly associated with gut dysbiosis and increased intestinal permeability, may be further exacerbated by elevated endogenous ethanol levels—particularly in the context of impaired hepatic ethanol metabolism. This suggests a potential vicious cycle in which gut-derived ethanol perpetuates liver injury and disease progression.

Given the increasing global burden of MASLD, and the emerging evidence implicating microbial ethanol production in liver pathology, it may be clinically relevant to screen high-risk patients—especially those with unexplained disease progression or elevated blood ethanol levels despite abstinence—for the presence of alcohol-producing gut microbiota. Microbiota-targeted interventions, including the modulation or selective eradication of ethanol-producing species, represent a promising yet largely unexplored therapeutic strategy in this context.

Nonetheless, ABS remains a rare entity, with limited epidemiological data. Although case reports have documented its coexistence with chronic liver disease (e.g., [1,27]), statistically robust associations have not yet been established. Well-designed prospective clinical studies are therefore urgently needed to elucidate the prevalence, pathogenic mechanisms, and clinical implications of endogenous ethanol production in chronic liver disease—and to assess whether targeted microbiome modulation may offer therapeutic benefits.

## Data Availability

The data presented in this study are available on request from the corresponding author.

## Abbreviations

The following abbreviations are used in this manuscript:

ABS	autobrewery syndrome
ACC1	acetyl-CoA carboxylase 1
ADH	alcohol dehydrogenase
AJs	adherens junctions
ALD	alcoholic liver disease
ALDH	aldehyde dehydrogenase
AMPK	adenosine monophosphate kinase
CD36/FAT	fatty acid translocase
COL1A2	collagen type I alpha 2 chain
CYP2E1	cytochrome P450 2E1
CYP2E1	cytochrome P450 family 2 subfamily E member 1
ECM	extracellular matrix
EnEth	endogenous ethanol
ERK1/2	extracellular signal-regulated kinase ½
EtG	ethyl glucuronide
EtS	ethyl sulfate
FAEEs	fatty acid ethyl esters
FASN	fatty acid synthase
FATPs	fatty acid transporters
FFAs	free fatty acids
GMT	fecal microbiota transplantation
HSCs	hepatic stellate cells
IL-1β	interleukin-1β
LPIN	lipin1 protein
LPS	lipopolysaccharide
MAPK	mitogen-activated protein kinase
MASH	metabolic associated steatohepatitis
MASLD	metabolic-associated steatotic liver disease
MCJ	methylation-controlled J protein
MEOS	microsomal ethanol oxidizing system
MLCK	myosin light chain kinase
MMP-1, MMP-2	matrix metalloproteinase-1, 2
n-3 PUFA	omega-3 polyunsaturated fatty acid
NA	non applicable
NAD	nicotinamide adenine dinucleotide
NADH	nicotinamide adenine dinucleotide (NAD) + hydrogen (H)
NAFLD	non-alcoholic fatty liver disease
NF-κB	nuclear factor-kappa B
NO	nitric oxide
NRF2	nuclear factor erythroid 2-related factor 2
p70S6K	P70-S6 kinase 1
Peth	phosphatidylethanol
PI3K	phosphatidylinositol 3-kinase
PPARα	peroxisome proliferator-activated receptor alpha
PTPase	protein tyrosine phosphatase
Reg3	regenerating islet-derived protein 3 gamma
ROS	reactive oxygen species
SCD1	stearoyl-CoA desaturase 1
SIRT1	sirtuin 1
SMAD3	mothers against decapentaplegic homolog 3
SREBP-1c	sterol regulatory element-binding protein 1c
TBARS	thiobarbituric acid reactive substance
TGF	tumor growth factor
TJs	tight junctions
TLR/	toll-like receptor 7
TLR4	toll-lik receptor4
TNF-α	tumor necrosis factor alpha
ZO-1	zonula occludens 1
